# Sodium-induced population shift drives activation of thrombin

**DOI:** 10.1038/s41598-020-57822-0

**Published:** 2020-01-23

**Authors:** Ursula Kahler, Anna S. Kamenik, Johannes Kraml, Klaus R. Liedl

**Affiliations:** Institute of General, Inorganic and Theoretical Chemistry, University of Innsbruck, Innrain 82, 6020 Innsbruck, Austria

**Keywords:** Computational models, Computational biophysics

## Abstract

The equilibrium between active E and inactive E* forms of thrombin is assumed to be governed by the allosteric binding of a Na^+^ ion. Here we use molecular dynamics simulations and Markov state models to sample transitions between active and inactive states. With these calculations we are able to compare thermodynamic and kinetic properties depending on the presence of Na^+^. For the first time, we directly observe sodium-induced conformational changes in long-timescale computer simulations. Thereby, we are able to explain the resulting change in activity. We observe a stabilization of the active form in presence of Na^+^ and a shift towards the inactive form in Na^+^-free simulations. We identify key structural features to quantify and monitor this conformational shift. These include the accessibility of the S1 pocket and the reorientation of W215, of R221a and of the Na^+^ loop. The structural characteristics exhibit dynamics at various timescales: Conformational changes in the Na^+^ binding loop constitute the slowest observed movement. Depending on its orientation, it induces conformational shifts in the nearby substrate binding site. Only after this shift, residue W215 is able to move freely, allowing thrombin to adopt a binding-competent conformation.

## Introduction

The serine protease thrombin plays a key role in the blood coagulation cascade as it catalyses the cleavage of fibrinogen, which ultimately leads to the formation of blood clots^1–4^. Due to its central role for the blood coagulation, it is a compelling drug target and its structure has been examined thoroughly, with the first X-ray structure published in 1989^5^.

Thrombin is fully active in the presence of Na^+^, leading to the description of thrombin as allosterically regulated by Na^+^ binding^6^. This leads to the common distinction between ‘slow’ Na^+^-free, and ‘fast’ Na^+^-bound thrombin^7^. While the fast form cleaves fibrinogen and protease-activated receptors, the slow form is more specific towards protein C, imparting an anticoagulant effect to it^8^. The ratio between fast and slow forms was estimated to be 3:2 at physiologic temperature and salt concentration^8,9^. Na^+^ binds over 15 Å away from the catalytic triad, embedded in the Na^+^ binding loop and coordinated by two backbone carbonyl oxygen atoms and four buried water molecules^10,11^.

**Figure 1:**
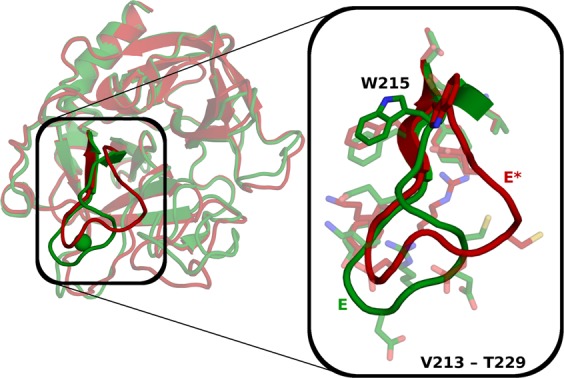
Structural comparison between active and inactive forms of thrombin. The active E form (depicted 3LU9, green) and the inactive E* form (depicted 3BEI, red) mainly differ in the bottom of the non-prime site and the Na^+^ binding loop. The detailed view shows the loop chosen for further analyses (V213–T229).

Based on stopped-flow fluorescence measurements, a two-step mechanism of thrombin activation has been proposed^9,12^. The active form E can bind to Na^+^ to build the fully active E:Na^+^ form, while the E* form cannot bind Na^+^. Na^+^ binding stabilizes the E form and thus shifts the equilibrium towards active thrombin^9,13,14^. Under physiological conditions the E* form is barely present^9^ but it can be stabilized in mutants. Thus, X-ray structures of E* often feature mutations^15,16^, which allow structural interpretation of their observed catalytic inactivity. E* structures typically display a disarrayed conformation of substrate binding site residues W215−E217, which hinders substrate binding. Particularly, the sidechain of W215 does not build the bottom of the S3 pocket as in the E form, but instead assumes an upright orientation and blocks the binding cleft (Fig. 1). From the residues W215−E217, the structural differences extend to nearby protein regions, i.e., into the Na^+^ binding loop.

While active and inactive forms can be distinguished based on their structure, the mechanism of the transition between the forms and influencing factors are not fully understood. The presence or absence of Na^+^ in the X-ray structure models is often unreliable as Na^+^ ions and water molecules have an equal number of electrons and are thus difficult to distinguish^11^.

Spectroscopic measurements^17^ and NMR measurements^18–20^ showed that apo thrombin is highly flexible, especially in the surface loops. It is rigidified upon binding to a substrate or Na^+^, in particular in regions around the substrate binding site. This further supports the assumption that apo thrombin exists as a highly diverse ensemble including the active as well as inactive and zymogen-like conformations^17,18,21,22^.

Substantial scientific effort has been dedicated to characterize this ensemble of thrombin conformations and to identify factors that bias it towards active or inactive states. For example, kinetic studies have been performed to characterize the E and E* distribution. It was found that the E form is more favourable and that both forms interconvert on a timescale of several milliseconds^23–25^. The most prominent factor biasing the probabilities in the conformational ensemble of thrombin is, as mentioned above, the presence of Na^+^ ions. Several experimental studies show that binding of a Na^+^ ion selects an open conformation and thus shifts the equilibrium towards the stabilized, proteolytic-active enzyme^17,18,21,22^. Additional computational studies were able to provide further insights on the structural consequences of Na^+^ binding. De Amorim *et al*.^26^ compared molecular dynamics (MD) simulations of thrombin with and without the Na^+^ ion bound and found that the Na^+^-free form adopts a more closed conformation of the binding site. Later studies, also address the influence of Na^+^ association^27,28^ and observe a stabilization of the conformation with an open substrate binding site in presence of Na^+^^29^.

Besides Na^+^ binding, several other factors have been observed to influence the equilibrium between active E and inactive E* forms. Pozzi *et al*.^30^ proposed the electrostatics at the W215−E217 segment as key factor, since mutations that stabilize the E* form predominantly lower the negative charge. Peacock *et al*.^31^ focused specifically on effects caused by mutations of the residues W215 and F227. Using activity assays, hydrogen-deuterium exchange experiments and accelerated MD simulations, they found that the π-stacking interactions between the two residues are essential to maintain an optimally active conformation. Residue W215 was found to be allosterically connected to the Na^+^ binding loop and the active site.

Melge *et al*.^32^ investigated functional impacts of the Na^+^ binding loop using MD simulations to study the mutation R221aQ (chymotrypsin numbering) in prothrombin, which causes thrombosis. They hypothesized that the dysfunction originates from the change of electrostatics in the Na^+^ binding loop. The mutation R221aQ is assumed to strengthen the affinity to Na^+^ and thereby to shift the equilibrium of thrombin to the active E form.

Further computational studies were employed to investigate the relation of function and dynamics of thrombin. Fuglestad *et al*.^33^ and Gasper *et al*.^34^ used conventional and enhanced sampling to study correlated motions, community networks and influence of allosteric binding of thrombin. They found complex coupled motions between the active site and distant exosites taking place on slow timescales. In another study by Wu^35^, conformational changes along the transition between the E and the E* form were monitored with targeted molecular dynamics (TMD) simulations. Using this enhanced sampling method, clusters of hydrophobic residues near the substrate binding site were identified, which undergo drastic conformational changes. The importance of the varying flexibility of proteases was further highlighted by Fuchs *et al*.^36^ In their study on thrombin, a clear connection between dynamics in the binding site and substrate recognition was observed. Altogether, over years of research, numerous experimental and computational studies of thrombin have accumulated.

Besides thrombin, other proteases of the chymotrypsin fold also show equilibria between active and inactive forms that parallel the E − E* equilibrium of thrombin, e.g., complement factor D, chymotrypsin and tryptase were crystallized in the E* form^15,16^. MD simulations of trypsin showed that it exists in a variety of states, both active and inactive^37^. The equilibrium between E and E* also extends to zymogen forms of the proteases, although the equilibrium is shifted in favour of the inactive E* form^23,38^.

In this work, we aim at gaining a better understanding of the dynamics involved in the equilibrium between active and inactive forms of thrombin in atomic detail. With enhanced sampling we cover a broad ensemble, which expands the conformational space that is known from crystallographic structures. We build Markov state models (MSMs)^37,39–43^ from simulations with and without Na^+^ ions to compare thermodynamic and kinetic properties. From several structural rearrangements that are necessary for a complete transition between E and E* forms, we identify the slowest conformational change and shed light on the mechanism of the activation.

## Results

### Sampling transitions between E and E*

X-ray structures show different structural changes that render the E* form inactive (closing of the binding cleft by reorientation of residue W215, shift of the W215–E217 loop to close the S1 pocket). They are accompanied by a twist of the Na^+^ binding loop (Supplementary Figs. S1 and S2). However, the relation of the rearrangements and the influence of Na^+^ cannot be fully understood by analysis of static X-ray structures alone.

To understand the mechanism of the transition itself and to gain information about the timescales of the conformational changes involved in the transition, we performed and analysed MD simulations. Classical MD (cMD) simulations of 1 µs length started from the E and the E* form extend the conformational space covered by X-ray structures. However, no full transition between the forms can be sampled in that simulation time (Supplementary Fig. S3).

To sample the complete transformations between E and E*, we perform TMD simulations that enforce transitions by adding a distance-dependent bias potential. Simulations are started from an E structure and forced to E* and vice versa. In the course of the simulations, they approach the chosen target form.

The applied bias potential and methodology of TMD simulations does not allow drawing direct conclusions on the unbiased mechanism between active and inactive forms of thrombin. Hence, we used structural information on the transition pathway, extracted from the TMD simulations, to seed a large number of cMD simulations. Starting structures for these cMD simulations are thus distributed over a broad region of the conformational space and include high-energy states. While one set of simulations does not contain Na^+^ ions, another one was set up with Na^+^ ions present, matching the physiological blood concentration. The simulations are combined, using MSMs to extract thermodynamic and kinetic information.

### MSMs reveal population shift towards the active form in the presence of Na^+^

As a first step the coordinate reduction method time-lagged independent component analysis (TICA) is applied on the seeded cMD simulations with and without Na^+^. This analysis filters out coordinates that contribute to the slowest motions in the systems (Supplementary Fig. S4). Clustering in the TICA space results in discrete trajectories, which are then used to construct two MSMs, one from the simulations without Na^+^ and one from the simulations with Na^+^.

The big gap between the slowest and second slowest timescale points towards the existence of one process that is significantly slower than all others (Supplementary Fig. S5). Therefore, we chose to simplify the model with PCCA++ into two metastable states. It lumps microstates together that have a large transition probability. Chapman-Kolmogorow tests support the reliability of the MSMs with two metastable states (Supplementary Fig. S5).

**Figure 2:**
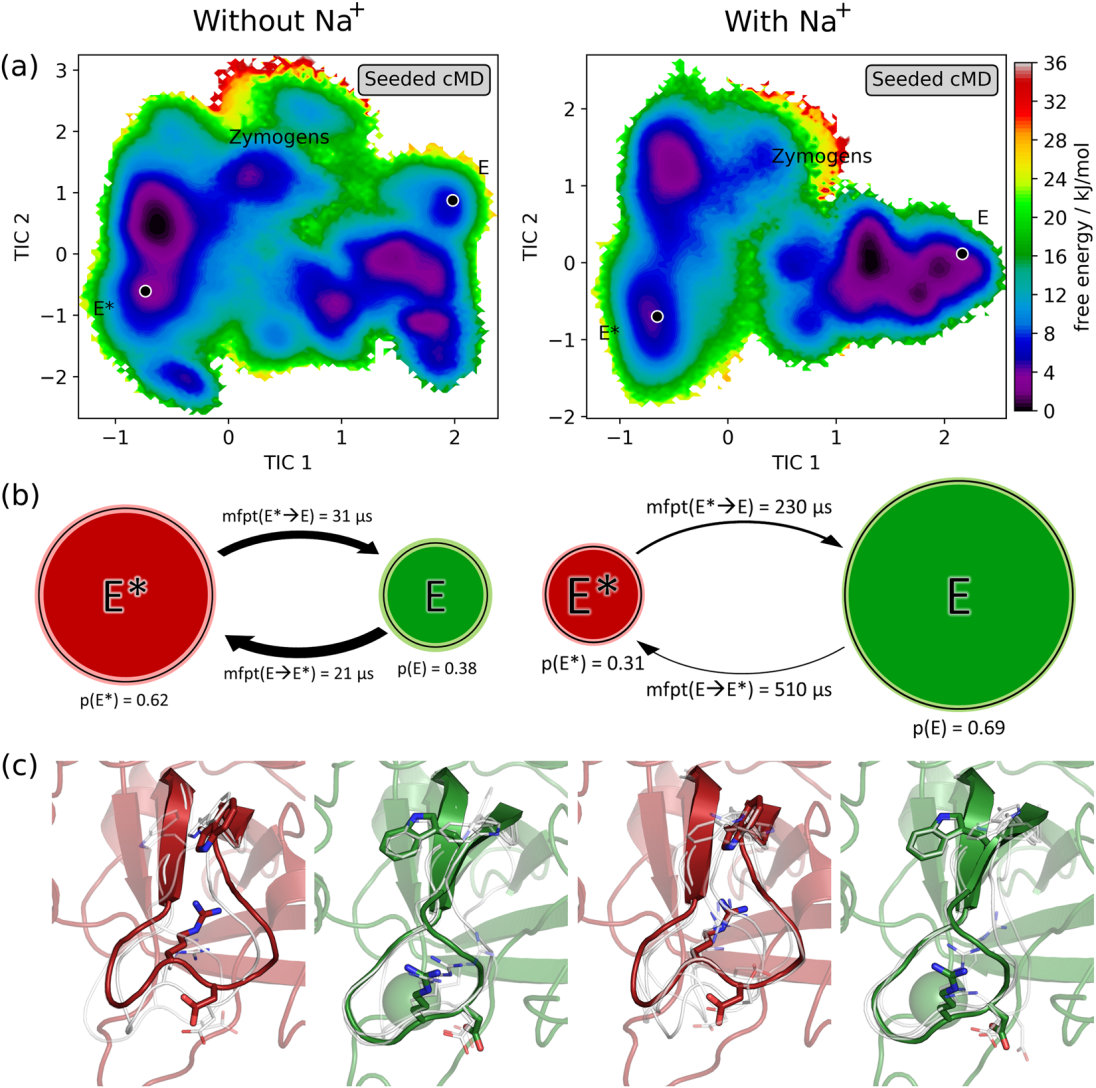
MSMs of thrombin without Na^+^ and with Na^+^ ions. (**a**) The free energy surface projected on the TICA space shows the shift of the populations depending on the presence of Na^+^. The position of the X-ray structures of the E form (3LU9) and the E* form (3BEI), from which the TMD simulations were started, are marked. (**b**) Calculated probability of E-like and E*-like states and mean first passage times (mfpt) for the transitions between the states. Brighter colors denote the confidence interval of the probabilities based on the uncertainties of the Bayesian MSMs, for errors of the mfpts please refer to the Supplementary Tables 1 and 2. (**c**) Comparison between three representative structures of the metastable states, E and E* (white), and an X-ray structure of the respective form (green and red).

The equilibrium probabilities of the microstates, calculated with the MSMs, can be used to weight the trajectory frames. Figure 2 shows the resulting free energy surface projected on the first two TICA coordinates. In the simulations without Na^+^ ions added, the most favourable free energy minimum is situated near to the minimum where the projected experimental structures of the E* form are found, separated only by a low barrier. The minima near the E form of thrombin are significantly shallower. In contrast, in the simulations where Na^+^ ions are present, the most favourable minima are situated near the X-ray structures of the E form, while the minima around E* are less pronounced.

These observations are reflected in the calculated probabilities of the two-state models (Fig. 2b). In the simulations without Na^+^, the most occupied state has a probability of 0.62 and includes conformations structurally similar to the E* X-ray structures (RMSD of residues V213–T229 to E* form X-ray: 0.77 to 5.85 Å). The less populated state has a probability of 0.38 and includes conformations that are similar to the E form (RMSD to E form X-ray: 0.56 to 5.82 Å). Vice versa, in the simulations with Na^+^, the E-like state has a higher probability of 0.69 (RMSD to E form X-ray: 0.54 to 5.02 Å) and the E*-like state only of 0.31 (RMSD to E* form X-ray: 0.71 to 6.02 Å). The distributions of RMSD values within the trajectories and within the metastable states are shown in Supplementary Fig. S6. The estimated mean first passage times (mfpts) between the states is considerable lower in the simulations without Na^+^ (21 µs and 31 µs respectively) than in the simulations with Na^+^ (230 µs and 510 µs). Confidence intervals for the calculated probabilities and mfpts are listed in Supplementary Tables S1 and S2.

Structurally, the metastable states are remarkably diverse (Fig. 2c). While they each contain conformations similar to the respective X-ray structure, they also include structures that seemingly have little in common with them. For example, the E state in the simulations with Na^+^ comprises conformations that are very similar to X-ray structures bound to substrate analogues but also conformations with a distorted binding site that renders the substrate binding impossible. However, despite their structural differences these conformations are kinetically close as the barrier between them is low (Fig. 2a) and the transition is comparably fast.

For a quantitative understanding of the activation mechanism, we establish four intramolecular features based on major structural differences in the X-ray structures (Supplementary Fig. S2). These features are chosen as they differ strongly between the X-ray structures of E and E* (Fig. 1 and Supplementary Fig. S1) and/or between the MSM states. In part these features have obvious implications on the ability of thrombin to bind substrates and have been discussed previously to distinguish E and E* forms^23^. To capture the orientation of the W215 sidechain, we measure its distance to the catalytic triad (stable internal position). In the X-ray structures of the E form, W215 is set at the bottom of the non-prime site (large distance to the catalytic triad), while in the E* form it takes an altered position close to the catalytic centre and occludes the substrate binding site (small distance to the catalytic triad). In the following we refer to this distance as *WCT*. Furthermore, we measure the distance between R221a, part of the Na^+^ binding loop, and the catalytic triad (will be referred to as *RCT* in the following). RCT varies strongly among the X-ray structures. In the E* form R221a faces upwards towards the S1 pocket (small distance to the catalytic triad) and in the E form it points into the solvent (large distance to the catalytic triad). Another prominent difference of the X-ray structures is the occlusion of the S1 pocket in the E* form by the loop W215−E217, which hinders binding of the substrate. To capture it, the distance between G193 and G216 is used (*GG*), since substrates bind between these residues. The distance GG is large in E and small in E*. The whole Na^+^ binding loop varies strongly between the E and the E* form. The torsion around the φ dihedral of residue D221 (*PhiD*) distinguishes the E form (negative values) and the E* form (positive values) in the X-ray structures as well as in the MSMs.

**Figure 3:**
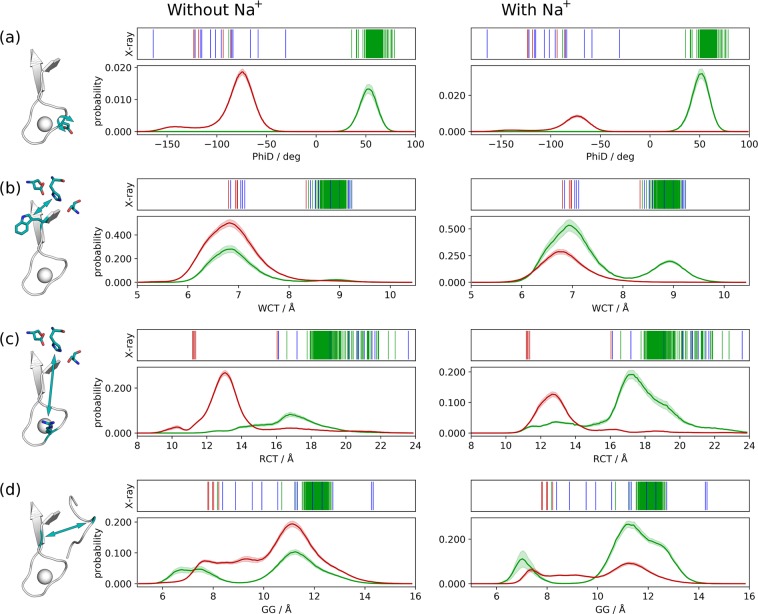
Distributions of internal distances in the E and the E* state, based on the MSMs without and with Na^+^. The frames are weighted according to the probabilities calculated from the MSMs, so that the combined area under both curves totals to 1. The distribution of (**a**) φ torsion of D221 (PhiD), (**b**) distance between W215 and the catalytic triad (WCT), (**c**) the distance between R221a and the catalytic triad (RCT) and (**d**) the distance between G193 and G216 (GG) in the E state (green) and the E* state (red) are displayed. The left column shows the results for the simulations done without Na^+^, the right column the results for simulation with added Na^+^. The panels above the distributions show the values for the features in X-ray structures.

The distributions of these features in the simulations with and without Na^+^ are shown in Fig. 3, weighted with the probabilities from the MSMs and separated into the E and the E* state.

PhiD gives a clear-cut distinction between the E and the E* states. In the simulations with Na^+^, large values are more likely, mirroring the shift of the equilibrium between the states. W215 is almost exclusively blocking the binding site if no Na^+^ is in the solution. Na^+^ promotes large values for WCT as it stabilizes the E state, which includes open conformations of thrombin. In the E* states of the MSMs, R221a is more likely buried within the Na^+^ loop and not pointing into the solvent, while in the E state the opposite is promoted. The orientation of R221a strongly correlates with PhiD, i.e., the dihedral of the neighbouring residue. GG does not so strongly depend on the present metastable state. The E and the E* form both contain conformations with open and closed S1 pocket, but the respective probabilities are different, resulting in a shift towards an accessible S1 pocket caused by Na^+^.

These distributions show that the Na^+^ binding loop, being involved in the slowest observed conformational change, influences the arrangement of the nearby substrate binding site. Especially the orientation of W215 and the accessibility of the S1 pocket are affected by the loop conformation and structurally determine thrombin’s ability to bind to substrates and thereby its activity. The distribution of the Na^+^ binding loop conformations depends on the presence of Na^+^ ions and in turn shifts also the equilibrium distribution of substrate binding site conformations.

## Discussion

Due to its central role in blood coagulation, thrombin is one of the most extensively studied serine proteases. Thrombin is known to exist as an equilibrium of active and inactive states. A multitude of comprehensive studies was dedicated to identify the decisive factors balancing this equilibrium (e.g., Na^+^ binding, exosite binding, mutations) and to profile effects of their perturbation. X-ray crystal structures of active E forms, inactive E* forms and zymogens as well as NMR measurements elucidate the structural origins of the different activities^14,21^.

The active form has been crystallized bound to various substrates, substrate analogues or inhibitors as well as in apo form, resulting in a multitude of very similar structures. X-ray structures of inactive forms allow to identify conformational differences to the active form. The mechanisms involved in the transition between the forms however cannot be explained by review of the structures alone and a dynamic approach is required to truly understand the associated conformational changes.

As the X-ray structures between the E and the E* form exhibit substantial variations, it is clear that major conformational rearrangements are necessary for activation and deactivation (Fig. 1). This implies that MD simulations would need to cover microsecond to second dynamics to sample the transitions^44^, which exceeds the possibilities of routine cMD simulations. Therefore, we perform an enhanced sampling method, TMD. By introduction of an additional bias potential, it encourages transitions to a known structure, which here facilitates the sampling of the pathway between E and E* forms. In different TMD simulations with altered starting velocities and going in both directions, similar conformational states occur and the path taken is comparable. We construe that the bias of the TMD simulations does not distort the pathway to a drastic extent.

To capture an unbiased ensemble of the full transition, we clustered TMD simulations and started classical simulations of 200 ns length from each cluster representative. As the TMD simulations cover the transition between E and E*, also our seeding structures are distributed along the transition pathway (Supplementary Fig. S4). Although complete transitions between E and E* only seldom occur and are unlikely to be observed in 200 ns simulations, partial transitions between intermediate states take place and the simulations can be combined to create an MSM. The resulting MSM can then be used to investigate thermodynamic and kinetic properties of conformational changes on much longer timescales. Enhanced sampling techniques previously have successfully been used to seed simulations for the construction of MSMs^45^, used to investigate G protein activation^43^ and antibody dynamics^46^.

To investigate the influence of Na^+^ on the equilibrium between active and inactive conformations, we seeded simulations with and without Na^+^ ions. Since thrombin is only fully active in presence of Na^+^,^6^ the mechanism of this activation and its influence on the conformational ensemble has been a long-standing pursuit of many theoretical and experimental studies on thrombin. NMR experiments show a rigidification upon Na^+^ binding, leading to the suggestion that the role of Na^+^ is not to induce the active conformation but to select and stabilize the conformation from a pre-existing equilibrium^18^. In line with this, Xiao *et al*. observed stabilization of regulatory regions and catalytic pocket and shifts in the conformational space upon Na^+^ binding in MD simulations of thrombin^29^.

These findings coincide with the results of the present study. While the simulations with Na^+^ sample structurally similar conformations than those without Na^+^, the MSMs reveal a shift of the conformational space towards active states in the simulations with Na^+^. As the applied methodology is based on enhanced sampling, the observed changes are larger and slower, compared to the conformational shift described by Xiao *et al*.^29^, which mostly covers sidechain rearrangements.

While in the MSM, constructed from the Na^+^-free simulations, the metastable state that contains E*-like conformations is more likely (E:E* = 62:38), this trend is reversed in the simulations with Na^+^ where the state with E-like conformations is clearly more favourable (E:E* = 31:69). This shift can explain the higher activity of thrombin in presence of Na^+^. Bah *et al*.^9^ measured the ratio between slow (Na^+^-free) and fast (Na^+^-bound) thrombin to be 2:3, while the inactive E* form was described as barely populated (<1%). This deviates from our estimated population of 0.31 for the E* state in the system with Na^+^ (Fig. 2). However, the observed trend that adding Na^+^ leads to an activation of thrombin agrees with experiment, and we note that an exact description of the Na^+^ influence cannot be expected due to inherent approximations in classical force fields.

Regarding the observed kinetics, we see slower transitions between E and E* states in the system with Na^+^ (230 µs/510 µs) than in the system without Na^+^ (31 µs/21 µs), a difference of about a factor of 10. Although the estimation of the kinetic properties is only possible within a wide error margin, the difference between the systems is still significant (Supplementary Tables S1 and S2). Thus, especially on short timescales, the Na^+^-free ensemble shows much higher dynamics. Kinetic experiments yielded transition timescales in the millisecond timescale for the binding of a substrate-analogon^23–25^. However, so far no experimental measurements have been conducted, which would allow a direct observation of the transition timescales between E and E* without a binding partner. As the presence of a substrate and other environmental deviations, such as temperature, strongly influence transition timescales between E and E* conformations, the currently available kinetic measurement might not be ideally comparable to the presented values.

**Figure 4:**
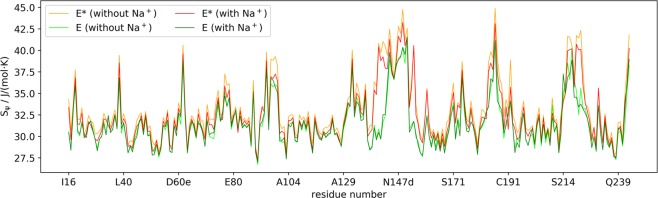
ψ entropies along the backbone of thrombin. The ψ entropies are a measurement for the localized flexibility. They are calculated for the structural ensemble of the E and E* metastable states from the MSMs without and with Na^+^.

In NMR experiments Lechtenberg *et al*. measured an ordering upon Na^+^ binding^18^, especially at the N-terminus, the 170s loop and the 180s loop. To compare the dynamics in the systems without and with Na^+^, we calculated dihedral entropies^36,47^ (Fig. 4). We observe lower flexibilities within the E state than in the E* state. The shift we observe in the equilibrium between the states caused by Na^+^ thus explains the rigidification in the NMR experiments. The regime of the calculated ψ entropies follows NMR observations and MD simulations^20,33^, which supports the reasonability of the applied approach.

Structurally, the conformations sampled in the Na^+^-free and the Na^+^-containing simulations are similar. The two metastable states for both MSMs comprise structures that are very similar to the respective E or E* X-ray structures but also conformations that differ significantly from them (Fig. 2). For example, the E state of the Na^+^-containing MSM consists of conformations that are nearly identical with the X-ray structure but also conformations that have a disrupted binding site and are thus unlikely able to bind and cleave a substrate. However, the barriers to catalytically active conformations within the metastable state are low and the transitions would occur fast — likely faster than substrate association. Following the concept of conformational selection^15,48^, substrates can select the active form from a larger ensemble.

The slowest transitions, i.e., the transitions between the metastable states, consist of a torsion and rearrangement of the Na^+^ binding loop, which is reflected by a clear splitting of the distribution of PhiD (Fig. 3). Other conformational rearrangements that are involved in the transitions between active and inactive states are faster and take place within the larger free energy minima, but are shown to correlate, in part strongly, with the loop conformation. As Na^+^ shifts the equilibrium between the metastable states, it also influences the distribution of other structural features that more obviously impact thrombin’s activity, e.g., W215 orientation (WCT) and opening of the S1 pocket (GG). Partially these conformational changes can already be observed within 1 µs cMD simulations, i.e., a closing of the binding cleft by W215 in Na^+^-free simulations of the E form and an opening of the S1 pocket in E* simulations (Supplementary Fig. S3). This corresponds to transitions over low barriers between neighbouring free-energy minima (Fig. 2). The much slower loop reorientation also affects the orientation of R221a (RCT), whose sidechain is directed into the Na^+^ binding site in known X-ray structures of E*. Thereby it compensates the positive charge of the missing Na^+^ ion. De Amorim *et al*. observe conformational changes as reactions to the displacement of Na^+^ in similar regions (loops at Na^+^ binding site, γ-loop, residues D189, W60d and W215) but in a less pronounced form^26^. Pelc *et al*. show that certain mutations in thrombin change the kinetic rate for transitions between E and E*. Based on their results, they conclude that interactions of W215, E217 and E192 play an important role in the stabilization of the E form and in the switch to E*.^25^ All of these residues are either part of the distinctive structural features we defined for this study, or in close vicinity.

Na^+^ almost exclusively binds in its native position, coordinated by R221a and K224, when PhiD is large (over 0°), i.e., in its E-form position. This is in contrast to Xiao and Salsbury^27^ finding the S1 pocket as predominant Na^+^ binding position. Most likely this is due to differences in the force field parameters and simulation setup.

On the basis of our observations, we propose that Na^+^ binding stabilizes the Na^+^ loop in its E conformation. The loop reorientation represents the slowest movement in the systems, making it the distinctive feature between the two metastable states E and E*. Other rearrangements that more intuitively influence the activity of thrombin, e.g., the orientation of W215 and the accessibility of the binding cleft for substrates, take place on a faster timescale, essentially constituting transitions within the larger minima. The Na^+^ loop orientation shifts the distributions of these characteristics, which changes the activity. W215 is locked in an upright orientation, when the loop is in an E*-like position. The switch of the Na^+^ binding loop to an E-like position gives the W215 additional freedom to open the binding cleft.

The E – E* equilibrium is not only present in thrombin, but has also been described in many other proteases of the chymotrypsin fold, prevalently in the zymogen form^15^. Plattner *et al*.^37^ found in MD simulations, analysed by the construction of an MSM, that in its apo state only small proportions of trypsin are present in the conformations known from its X-ray structures and able to bind the substrate. Other conformational states resemble E* conformations that are known from other related proteases, including the E* form of thrombin. Most related proteases lack thrombin’s Na^+^ binding ability and the corresponding loops are less distinct. However, length, conformations and mobility of these loops could still influence or even determine the properties of the particular E – E* equilibrium.

Our presented models promote the detailed mechanistic understanding of the E – E* equilibrium. This comprehensive description of the Na^+^ ion influence provides an intuitive explanation for the long standing debate on thrombin activation.

## Methods

### Structure preparation

The X-ray structures with PDB ID 3LU9^49^ and 3BEI^50^ were selected as starting point for MD simulations. The structures were prepared with MOE^51^. Everything but one heavy chain and the corresponding crystal waters was deleted. In 3LU9 thrombin was modelled back to its native sequence as it is rendered inactive in the structure by an S195A mutation. Likewise, the mutation D102N was reversed in 3BEI. The unresolved γ-loop was modelled with MOE. The structures were protonated with the protonate3D tool^52^ of MOE. To ensure consistency, the protonation was manually adjusted to the same protonation state for both systems. Water molecules of the TIP3P water model^53^ were added with LEaP of AmbertTools^54^, resulting in solvent boxes of 12 Å minimum wall distance. An extensive minimization and equilibration protocol was used to relax the systems^55^.

### TMD simulations

TMD simulations were performed using the plumed extension^56^ of GROMACS^57–59^. Transitions between 3LU9, representing the E form and 3BEI, representing the E* form, were enforced by applying a bias potential to conformations with an RMSD value deviating from the target RMSD. The equilibrated structure of the respective other form was used as reference for the calculation of the RMSD. The RMSD was calculated based on the heavy atoms of the protein after global alignment on Cα atoms. The target RMSD was lowered as a ramp from 2.3 Å to 1.0 Å during 100 ns of simulation and then kept constant for 40 ns. The bias potential was only applied when the momentary RMSD was larger than the target RMSD. The force constant of the harmonic potential was chosen to be 500,000 kJ/(mol·nm²).

### Seeding of cMD simulations

Ten TMD with different equilibrated starting structures and velocities, five in each direction, were clustered. Based on the heavy atoms of residues V213–T229, a hierarchical average-linkage algorithm^60^ was used on 14,000 frames, which were taken every 100 ps. From each of the 100 resulting representative cluster structures, three simulations were started: one with prior removal of solvent and a new equilibration with new water molecules added, one were both water molecules and Na^+^ ions were added (in physiological concentration of the blood) and one with the solvent directly taken from the TMD simulations without new equilibration. This resulted in 300 new cMD simulations, 200 without Na^+^ and 100 with Na^+^ ions.

These all-atom MD simulations were performed with pmemd^61^ in Amber^54^ using the Amber14SB force field^62^. The parameters for the Na^+^ ions were taken from Joung and Cheatham^63^. A uniform neutralizing plasma was used to neutralize the charges. A Van der Waals cutoff of 8 Å was applied. The Particle Mesh Ewald approach^64^ was used to deal with long range interactions. The systems were simulated in an NpT ensemble at a temperature of 300 K, kept constant by a Langevin thermostat^65^, and at 1 bar, regulated by a Berendsen barostat^66^. The SHAKE algorithm^67^ was used to restrain the hydrogen atoms and allow time steps of 2 fs.

Each of the new seeded cMD trajectories were run for 200 ns, resulting in a total of 60 µs simulation time.

Additionally, each 1 µs cMD simulations were performed, starting from the equilibrated X-ray structures of 3LU9 (both with added Na^+^ ions and Na^+^-free) and of 3BEI.

### PCA of experimental structures

Available structures of human thrombin were downloaded from the PDB^68,69^. 364 structures, those without missing atoms or mutations in residues V213–T229, were aligned based on the Cα positions. The Cartesian coordinates of heavy atoms of residues V213–T229 were used as input variables for a PCA, calculated with the Python package PyEMMA^70^. Classification into E, E* and zymogen was based on the corresponding literature. Snapshots from cMD every 100 ps and from TMD every 10 ps were extracted and projected on PC1 and PC2 of the X-ray PCA.

### TICA and MSM

Two separate TICAs^71,72^ were performed with PyEMMA^70^ for the Na^+^-free and the Na^+^-containing systems based on the seeded cMD simulation. From every 10 ps of these simulations, snapshots were extracted, totalling up to 4,000,000 and 2,000,000 used frames respectively. As input coordinates the residue-wise centres of mass of V213–T229 were used. For the Na^+^-containing simulations, additionally the binding of Na^+^ at the Na^+^ binding site was introduced as binary input coordinate that is only 1 when a Na^+^ ion is within 3.8 Å of both the backbone-oxygen of R221a and of K224.

The four components with the largest eigenvalue were used for kmeans clustering, setting the cluster number to 400. Bayesian MSM were constructed with the resulting discretized trajectories. A lag time of 80 ns was chosen as the estimated slowest timescales are approximately independent of the lag time at that point (Supplementary Fig. S6). At larger lag times, the loss of transition counts and emergence of unconnected states leads to discontinuities in the timescales.

### Analysis of the MSM

To simplify the MSMs, PCCA++ were performed, resulting in 2-state models as the timescale gaps between the slowest and second-slowest timescales are large. Stationary probabilities and mean first passage times between the states were calculated.

The four introduced distances that help to distinguish between active and inactive conformations and help to interpret the MSMs were evaluated with cpptraj of AmberTools^54^. WCT measures the distance between the centres of mass of the heavy atoms of W215 and the Cα atoms of the catalytic triad, RCT is the distance between the guanidinium C of R221a and the centre of the catalytic triad’s Cα atoms, GG is the distance between the Cα atoms of G193 and G216 and PhiD is the φ dihedral of residue D221.

To calculate the distributions of these features in the MSMs, we first identified the MSM microstates with a membership to a metastable state larger than 0.90 to eliminate states with uncertain affiliation. We then weighted the trajectory frames by the state probabilities that result from the MSMs. The error bars arise from the standard deviations of the state probabilities.

In order to select representative conformations, we separated the trajectory frames according to PCCA++ membership (again with a cut-off of 0.90), selected every 100 frame and clustered them structurally based on the RMSD of the heavy atoms of residues V213–T229. A hierarchical average-linkage algorithm was used to produce ten output clusters. The representatives of the three most populated clusters for both metastable states of the Na^+^-free and the Na^+^-containing systems have been displayed and compared to structures based on 3LU9 and 3BEI.

To capture the residue-wise flexibility of the separate states and systems, ψ entropies have been calculated for the beforehand selected frames, which were also used for the clustering^36,47^.

## Supplementary information

Sodium-induced population shift drives activation of thrombin.

## Data Availability

MD trajectories and datasets generated and analysed during the current study are available from the corresponding author on request.
