# Supplementary material for: Sodium-induced population shift drives activation of thrombin
Source: Sci Rep. 2020 Jan 23;10:1086. doi: 10.1038/s41598-020-57822-0 (PMC6978324; doi:10.1038/s41598-020-57822-0)
Supplement: Supplementary file 1 — Sodium-induced population shift drives activation of thrombin. [file 41598_2020_57822_MOESM1_ESM.pdf]

Supplementary Information:

Sodium-induced population shift drives activation of thrombin

Ursula Kahler, Anna S. Kamenik, Johannes Kraml, Klaus R. Liedl

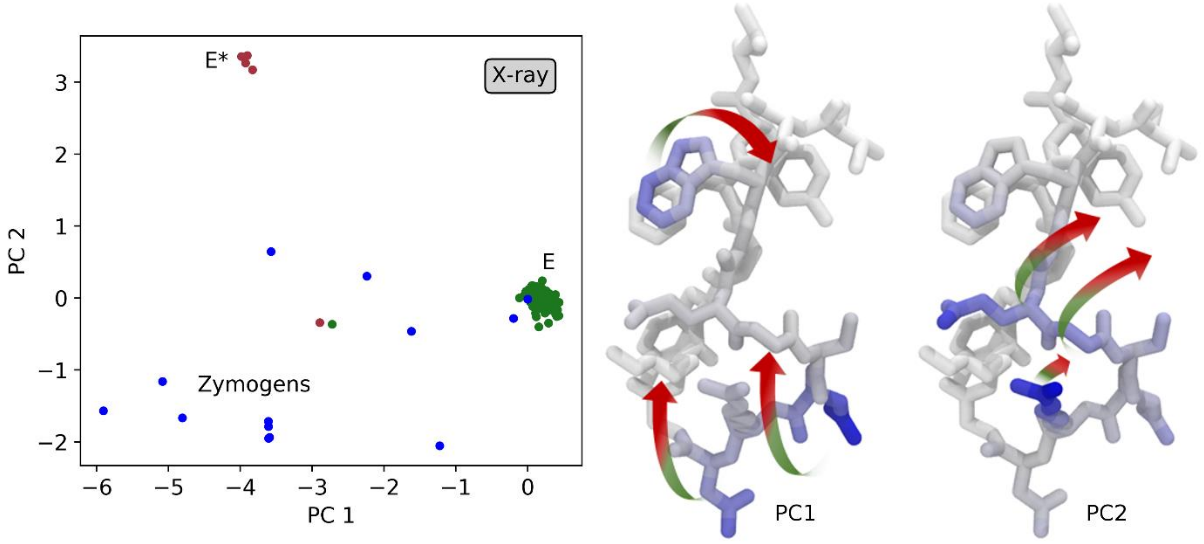

Supplementary Figure S1. PCA highlights structural differences among the X-ray structures. The first two principal components PC1 and PC2 separate the E form (green) and the E* form (red). Structures of zymogens (prothrombin and prethrombin, coloured in blue) scatter loosely in the region of low PC1 and low PC2 values. A structural interpretation for the PCs is shown on the right side. The blue colouring marks atoms that are involved in the PCs and the arrows schematically show the direction of the structural change.

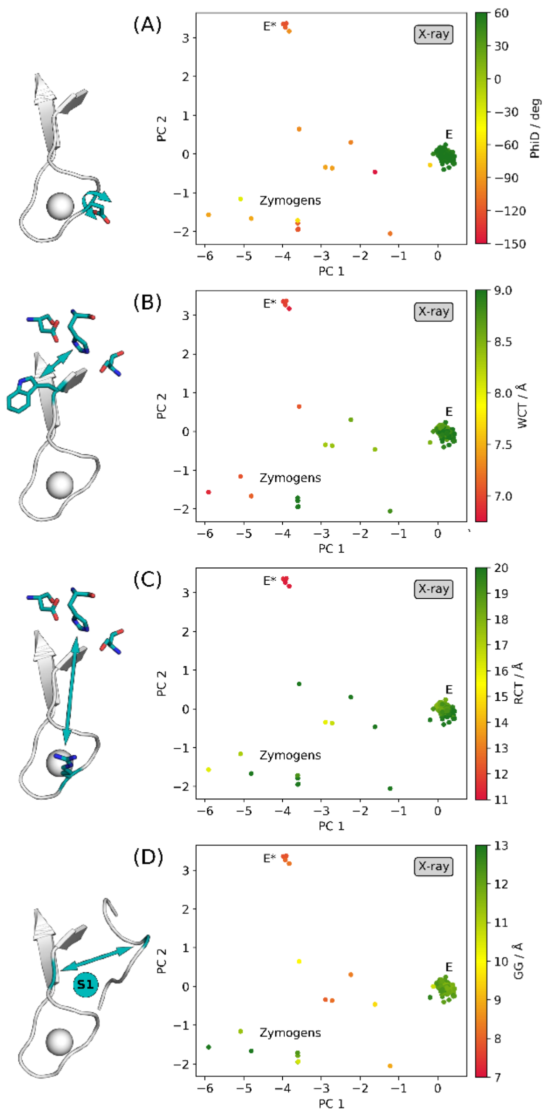

Supplementary Figure S2. Four features that discriminate between E and E* in X-ray structures. (A) The ϕ dihedral of residue D221 (phiD) is larger in the E form than in the E* form. (B) The distance between the centre of mass of the Cα atoms of the catalytic triad to the centre of mass of the heavy atoms of W215 (WCT) is larger in the active E form. (C) Residue R221a is oriented inwards in the E* form and leading to a shorter distance between its guanidinum C and the centre of mass of the Cα atoms of the catalytic triad (RCT). (D) In the E* form the S1 pocket is closed off by the W215−E217 loop manifesting in a shorter distance between the Cα atoms of G193 and G216 (GG).

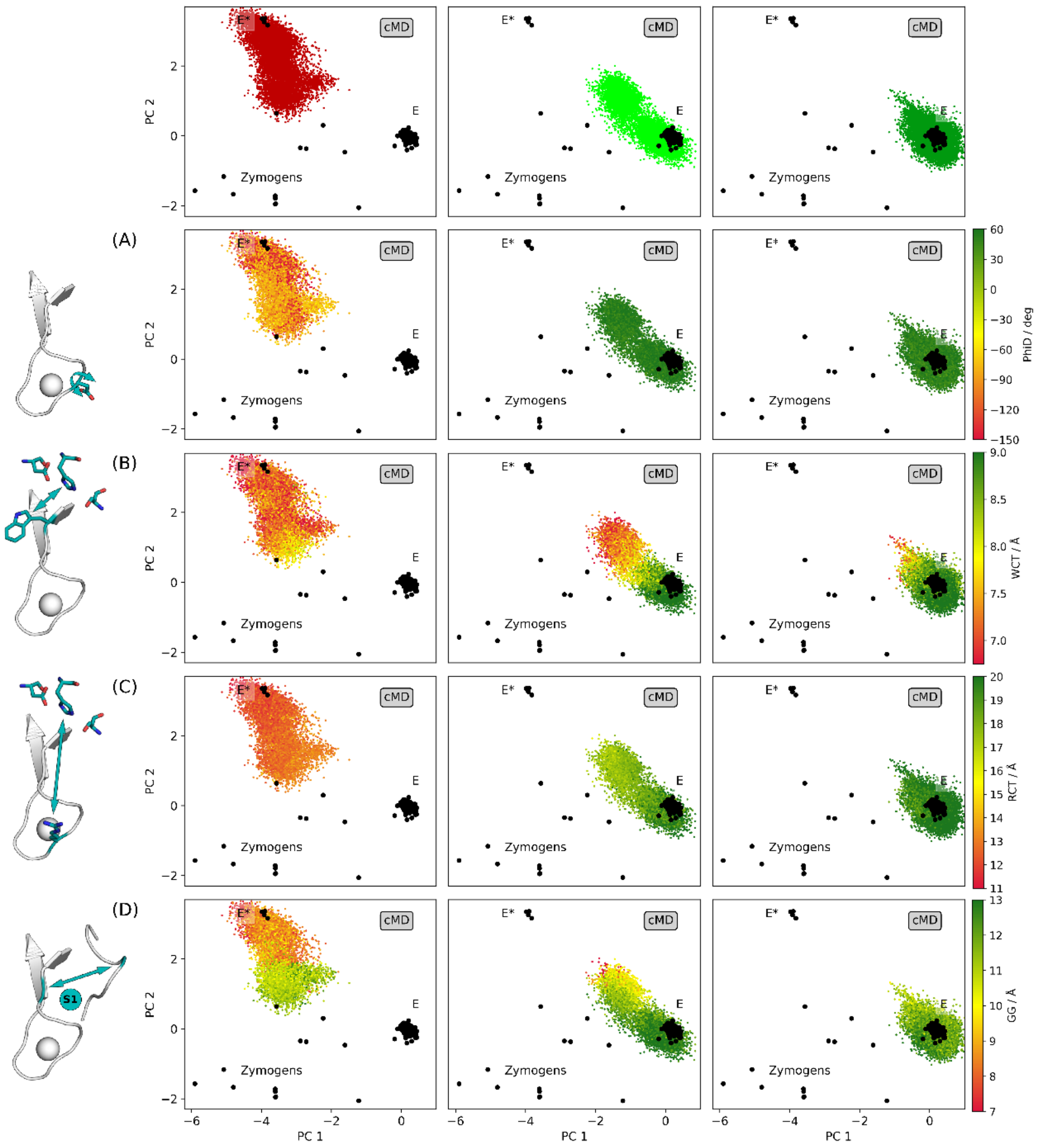

Supplementary Figure S3. Conformations of MD simulations projected on the X-ray PCA space. The experimental structures are depicted as black dots. Left: simulation started from the E* form (red in the top row), middle column: simulation started from E and without Na+ (light green in the top row) and right: simulation started from E with Na+ ions in solvent (dark green in the top row). Four structural features are calculated for the simulations: (A) the ϕ dihedral of D221 (phiD), (B) the distance between W215 and the catalytic triad (WCT), (C) the distance between R221a and the catalytic triad (RCT) and (D) the distance between G193 and G216 (GG).

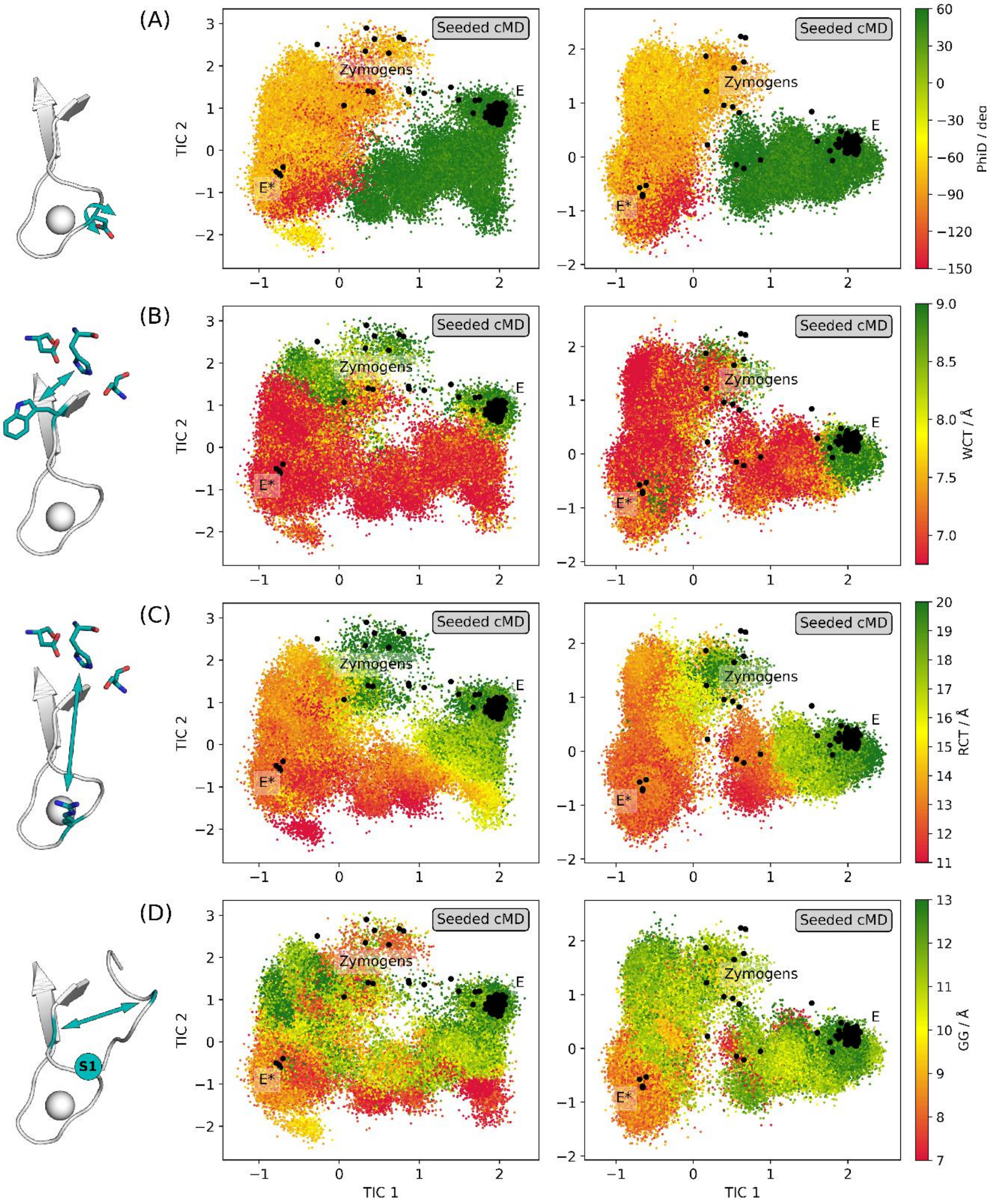

Supplementary Figure S4. Seeded simulations projected on TICA space and coloured according to internal features. The seeded cMD simulations shown on their TICA space, left: without Na+ and right: with Na+. The colouring corresponds to the calculated distances: (A) phiD, (B) WCT, (C) RCT and (D) GG. The X-ray structures are projected into this space as well and shown as black dots.

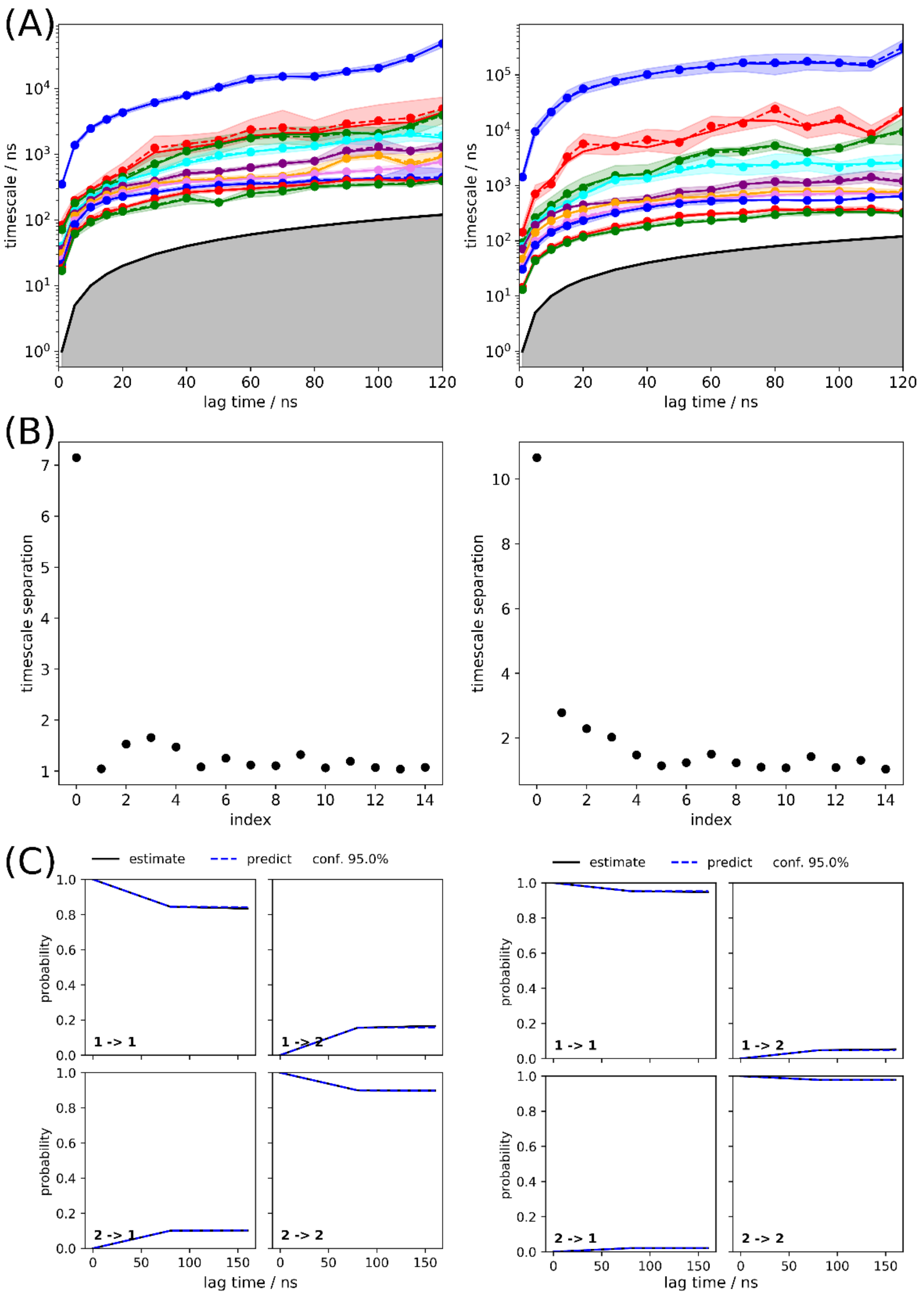

Supplementary Figure S5. Parameter selection and validation of the MSMs. Left: Simulations without Na^+, right: simulations with Na^+. (A) Based on the implied timescale plots, lag times of 80 ns were used. (B) The systems display one slow transitions between two metastable states. (C) Chapman-Kolmogorov plots support the reliability of the MSMs.

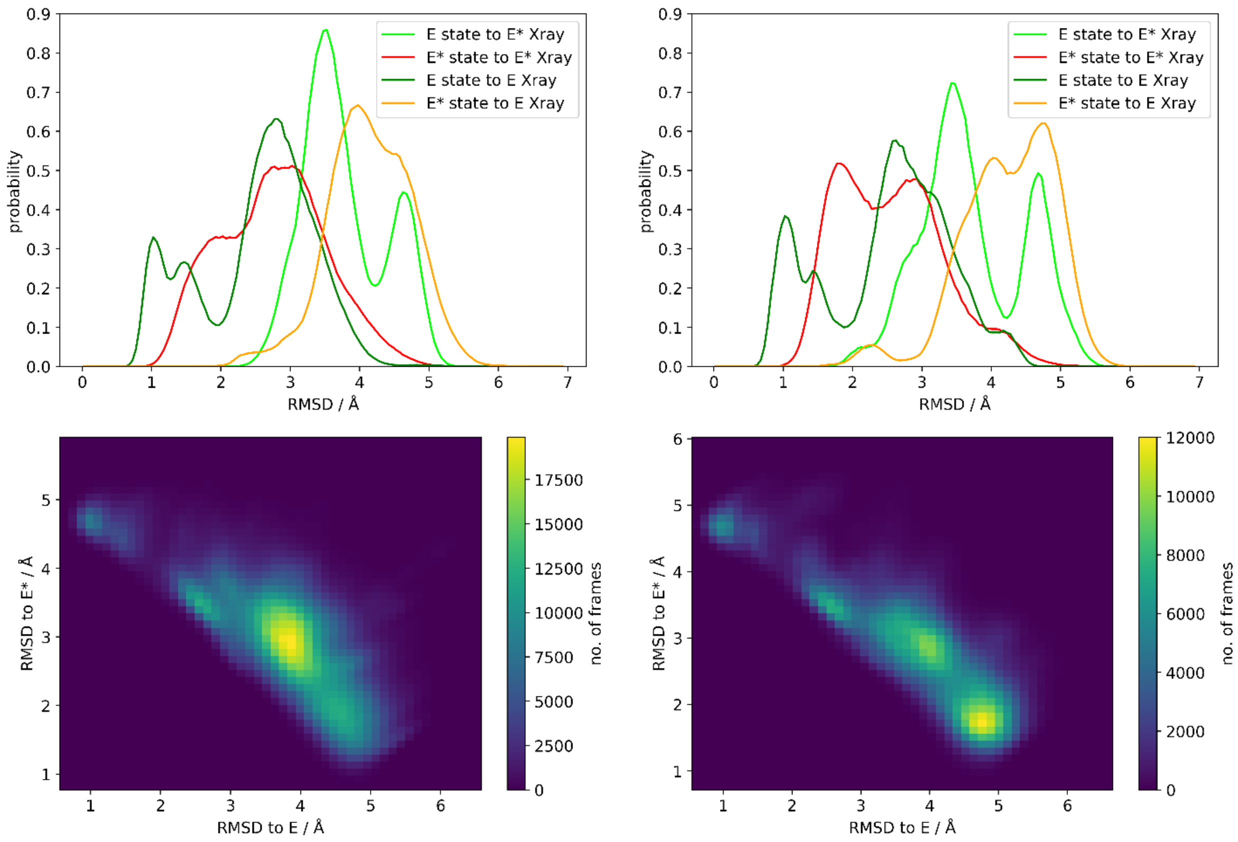

Supplementary Figure S6. RMSD to E X-ray (3lu9) and E* X-ray (3bei). The RMSD values are calculated of the heavy atoms of loop V213−T229 after alignment on all Cα atoms. Left: Simulations without Na^+, right: simulations with Na^+. Top: RMSD distribution within each metastable state to an E and an E* X-ray structure, normed to 1. Bottom: RMSD distribution within the trajectories without partition into states.

Supplementary Table S1. Mean values and confidence intervals for probabilities and mean first passage times calculated from the Bayesian MSM. The confidence intervals are calculated at a confidence level of 95%.

	without Na^+			with Na^+		
	mean	lower limit	upper limit	mean	lower limit	upper limit
p(E)	0.38	0.35	0.41	0.70	0.68	0.72
p(E*)	0.62	0.59	0.65	0.30	0.28	0.32
mfpt(E→E*) / µs	21	19	25	530	320	780
mfpt(E*→E) / µs	31	28	35	220	140	310

Supplementary Table S2. Mean values and confidence intervals for probabilities and mean first passage times calculated by omitting a 1/10 of the trajectories for each subsample. The analysis was performed on ten subsamples. The confidence intervals are calculated at a confidence level of 95%.

	without Na^+			with Na^+		
	mean	lower limit	upper limit	mean	lower limit	upper limit
p(E)	0.40	0.26	0.52	0.66	0.54	0.72
p(E*)	0.60	0.48	0.74	0.34	0.28	0.47
mfpt(E→E*) / µs	25	18	40	540	400	810
mfpt(E*→E) / µs	36	22	69	280	190	510
